# Correction: Matriptase Deletion Initiates a Sjögren’s Syndrome-Like Disease in Mice

**DOI:** 10.1371/journal.pone.0098331

**Published:** 2014-05-20

**Authors:** The PLOS ONE Staff

There is an error in the “Conclusions” section of the “Abstract.” The correct sentence for this section is:

"Our findings demonstrate that local impairment of epithelial barrier function can lead to loss of exocrine gland function in the absence of inflammation while systemic deletion can induce a primary Sjögren’s syndrome like phenotype with autoimmunity and loss of gland function."
